# Thirty minutes of daily artificial gravity does not mitigate head down tilt induced brain activity changes during cognitive task performance

**DOI:** 10.3389/fneur.2025.1602104

**Published:** 2025-08-27

**Authors:** Grant D. Tays, Heather R. McGregor, Yiri E. De Dios, Edwin Mulder, Jacob J. Bloomberg, Ajitkumar P. Mulavara, Scott J. Wood, Rachael D. Seidler

**Affiliations:** ^1^Department of Applied Physiology and Kinesiology, University of Florida, Gainesville, FL, United States; ^2^KBR, Houston, TX, United States; ^3^German Aerospace Center (DLR), Cologne, Germany; ^4^NASA Johnson Space Center, Houston, TX, United States; ^5^Norman Fixel Institute for Neurological Diseases, University of Florida, Gainesville, FL, United States

**Keywords:** spaceflight, cognition, artificial gravity, head down tilt (HDT) bed rest, fMRI

## Abstract

**Introduction:**

Studies have shown that microgravity results in high dual task costs when crewmembers perform cognitive-motor dual tasking. Head-down tilt bedrest (HDBR) has been widely used as a spaceflight analog environment, recreating some of the sensorimotor and cognitive changes, headward fluid shifts, and unloading of bones and muscles. Here, we examined whether artificial gravity (AG) mitigates changes in cognitive performance and associated brain activity that occur in the HDBR environment.

**Methods:**

We tested one group of participants (*n* = 16) that received 30 min of daily AG (half received it continuously while the other half experienced it in 5-min bouts), and one group that did not (*n* = 8 controls) during the course of 60 days HDBR. Participants performed spatial working memory and cognitive-motor dual tasking prior to entering HDBR, during HDBR and post-HDBR.

**Results:**

Brain activation patterns associated with these two tasks changed with HDBR, but there was no difference between the AG and control groups. Compensatory brain-behavioral change-change correlations were observed, where those who increased activation the most had the least decrease in motor tapping accuracy from pre-HDBR to late-HDBR.

**Discussion:**

These results suggest that AG does not reduce the need for compensatory brain responses that occur with HDBR, but longer duration and/or more optimal AG phasing may be required.

## 1 Introduction

Long duration spaceflight missions to the International Space Station (ISS) have widespread effects on human neurophysiology and performance. Sensory and motor deficits have been identified post-flight (1–13), and in-flight (14, 15). Cognitive changes due to spaceflight have been more difficult to identify.

Studies have reported decreased attentional capacity, decreased dual task performance, decreased spatial awareness, impaired performance on a visuospatial task, and complaints of “space fog” in microgravity (14, 16–22). Dual task performance has been shown to suffer when crewmembers are initially exposed to microgravity, but it then recovers over the course of weeks to months inflight (19, 20), suggesting that some manner of adaptation occurs. Similarly, deficits in dual-tasking postflight have been observed soon following return to Earth but recovers during the first week following long-duration missions (23). In the NASA Twins Study, the twin that went to space displayed more risk taking on the balloon analog risk test (BART; a decision making test to assess risk-taking behavior) during the entirety of his 1 year in spaceflight and also showed decreased task speed in the emotion recognition task and the digit symbol substitution task, as well as a decline in abstract matching (17). However, in a previous study we also analyzed digit symbol substitution task performance changes from pre-to postflight, finding no effects of microgravity in a larger cohort of astronauts (*n* = 15) that spent 6–12 months on the ISS (6). Further, we found no changes in spatial working memory performance from pre-to post-flight, but we did see alterations in brain functional connectivity (24) during task performance from pre-to postflight. This suggests that performance may be preserved with recruitment of additional compensatory neural resources post-flight, similar to what we identified post-flight in a sensorimotor adaptation task (25). In sum, some studies show cognitive performance changes with spaceflight whereas others do not. These inconsistencies could be due to a variety of factors including small sample sizes, individual differences, varying flight durations, times tested relative to g-transitions, varying tasks and cognitive domains assessed, and different task difficulty levels across studies.

We have previously assessed the effects of HDBR on cognition, finding increased performance on the card rotation task due to task practice (26). Further, we found decreased functional MRI activity during a spatial working memory task (SWM) within the right inferior frontal gyri and the left dentate nucleus. Moreover, participants that increased activation within the right angular gyrus increased spatial working memory performance, and those with a greater decrease in the inferior frontal gyri showed less declines in SWM accuracy (27). Further investigations have identified a general slowing in cognitive processing due to HDBR, particularly in sensorimotor processing speed (28, 29). Overall, these findings illustrate that even though cognitive performance may be maintained, there may still be underlying brain changes occurring with HDBR. These brain changes may reflect compensation or decreased neural efficiency as a result of HDBR. Recruitment of additional neural resources to maintain task performance levels may reduce the ability for dual tasking or to direct attention elsewhere.

HDBR involves adaptation to a new environment and sensory reweighting due to reduced foot sole and other somatosensory inputs and rotation of the body relative to the gravitational vector (30). Due to this, HDBR serves as a model environment to test integrated countermeasures such as artificial gravity (AG). This can be applied by having participants lay at the end of a short arm centrifuge spinning at a constant angular velocity. During the NASA Artificial Gravity Bed Rest – European Space Agency (AGBRESA) campaign, this velocity was adjusted for participant height and applied such that there was 1Gz at the center of mass (CoM), 2Gz at the feet and 0.3Gz at the head. AG is applied along the long axis of the body, increasing proprioceptive and somatosensory inputs, and stimulating the vestibular system, resulting in improved orthostatic tolerance relative to HDBR without AG (31–33). In a 5 day HDBR pilot study, AG reduced orthostatic intolerance with 30 min of daily AG (33). AG also reduced orthostatic intolerance in the 60 day AGBRESA campaign (76). We found that AG participants in AGBRESA performed better on the paced auditory serial addition task (PASAT, performed during active centrifugation) compared to control participants who performed the task in HDBR with no centrifugation, while other cognitive tasks showed no effects of AG (34). This was further supported by work showing learning effects (faster reaction time and increased accuracy) during simple and complex visual and auditory tasks accompanied by changes in electrocortical responses that may be related to impaired attentional processing, however, there were no counteractive effects of AG (35). Another study identified an overall slowing of cognitive performance across the AGBRESA campaign, including prolonged time to identify facial expressions during HDBR and an overall decrease in sensorimotor processing speed; these negative effects were not mitigated by AG (29).

Our primary aim was to examine whether AG applied along the long axis of the body via centrifugation would mitigate HDBR-induced changes in cognitive performance and associated brain activity. We administered the same spatial working memory and motor-cognitive dual task tests that we have previously used in our prior HDBR and spaceflight studies (26, 36–44, 77, 78). We found that during HDBR, there was increased functional brain activation during dual tasking across a large network containing frontal, parietal, cingulate, temporal and occipital cortices (45). In another HDBR study combined with elevated CO_2_ we found increased dual task cost effects in the middle temporal gyrus (78). We also observed decreased activation in the right middle frontal gyrus and left cerebellum while participants performed a SWM task in HDBR. Moreover, participants that improved SWM performance decreased their brain activity throughout HDBR, suggestive of practice effects (27).

Cognitive and sensorimotor declines that occur in HDBR and microgravity such as those that we have described here have been argued to stem from disrupted sensory inputs, sensory reweighting, and interference from adaptation to the microgravity environment. This study is a continuation of our previous efforts (34) to investigate the use of AG to counteract cognitive and sensorimotor brain and behavior deficits due to HDBR. In that study, we assessed overall behavior along the timelines as here and found that AG had minimal effects of cognitive performance, however, those that received AG had better performance on the PASAT, while other cognitive measures were largely unaffected. Functional mobility and balance performance showed significant effects due to HDBR, but not AG, but there was a large difference between those that received AG and those that did not in balance performance (34). Here, we hypothesized that those receiving daily AG would have reduced changes in brain activity during dual tasking during HDBR as compared to controls (in HDBR but receiving AG). Moreover, we predicted that this decreased activation would be associated with better cognitive performance. This would be suggestive of greater neural efficiency in the AG group.

## 2 Materials and methods

### 2.1 Participants

**Figure 1:**
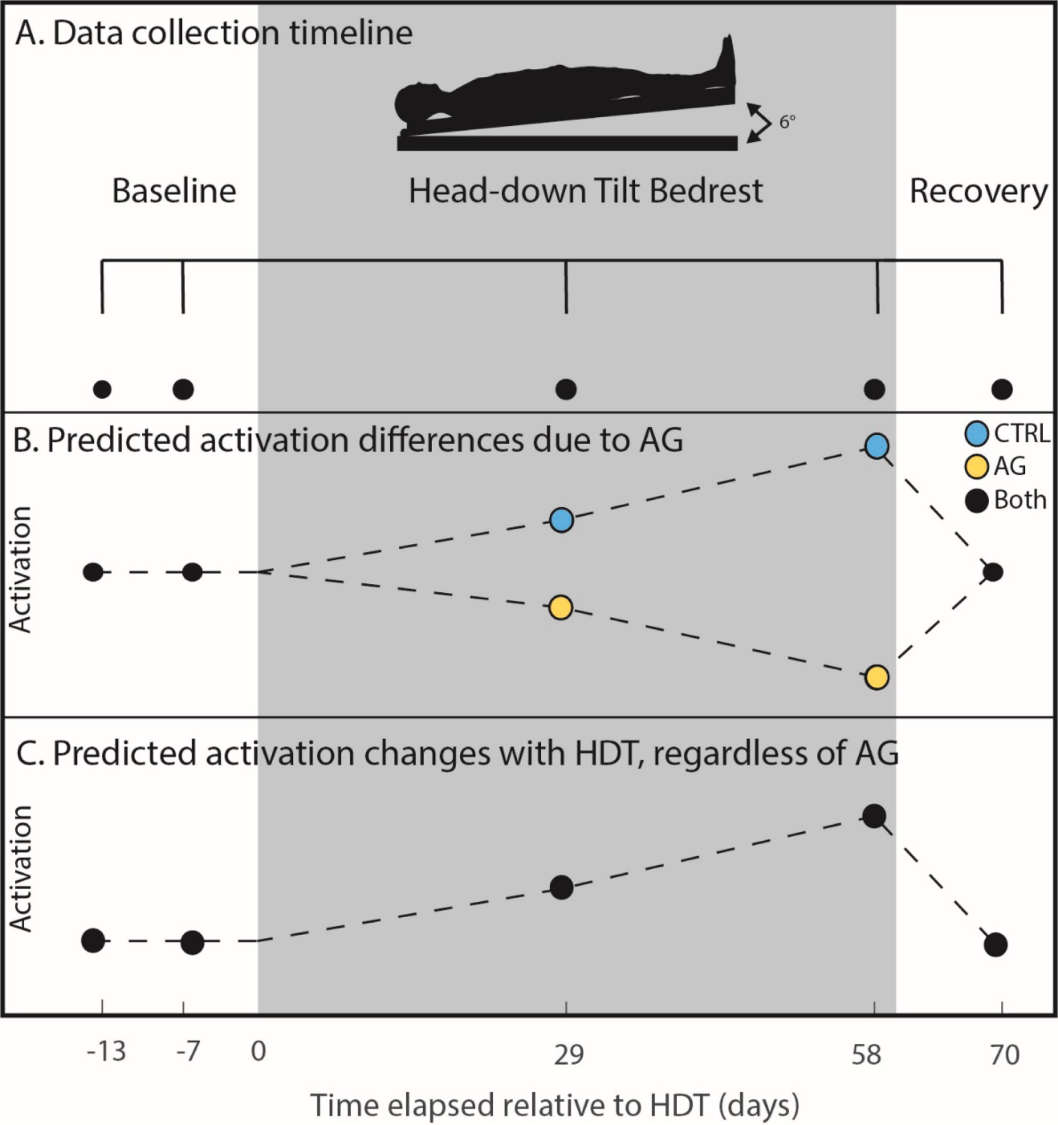
Cognitive task timeline. **(A)** Cognitive performance data were collected twice pre- (−13 and −7 days), twice during (29 and 58 days) and once post-HDBR (+10 days). Black dots indicate when data was collected for all individuals. The gray box extending across the figure from day 0 to day 60 represents the HDBR period in which all participants were head down tilt; the AG group received centrifugal AG for 30 min a day during this time. **(B)** Predicted activation patterns due to AG. We hypothesized that the CTRL group would experience increased functional activation while performing the task due to HDBR (causing interference), whereas the AG group would show decreased brain activation (i.e., increased neural efficiency). **(C)** In the case that AG would not have an effect, we also tested for increases in functional activation for all participants.

There were 24 (8F, 33.3 ± 9.17 yrs., 174.6 ± 8.6 cm, 74.2 ± 10.0 kg) participants that volunteered for this study and were assigned to one of three groups. The first two groups received daily centrifugal artificial gravity applied either (1) continuously in one 30-min bout daily (cAG); or (2) intermittently in 6 bouts of 5 min with 3 min rest in between (iAG). We established that there were no significant differences in brain or behavior changes (*p* > 0.05) (34) between the iAG and cAG groups, and thus pooled them into one group (AG) to increase our statistical power. The other group received no artificial gravity but were in HDBR (control group, CTRL). All subjects were familiarized with AG twice (BDC-11 and BDC-4) during the baseline phase, prior to being separated into groups. Participants were screened for tolerability through a tolerance test (AG2 protocol) to ensure they would be able to complete centrifugation. They were also selected to be similar in age, sex and education level to astronauts. The University of Florida and NASA Institutional Review Boards as well as the local ethical commission of the regional medical association (Ärztekammer Nordrhein) approved all study procedures. Informed consent was obtained from all participants. Participants first underwent 14 days of baseline data collection prior to entering HDBR, followed by 60 days of 6° HDBR and then 14 days post-HDBR (Figure 1). They performed a wide range of neurocognitive tasks including the paced auditory serial addition task, digit symbol substitution task, rod and frame task, and sensorimotor tasks four times, 7 days prior to entering HDBR, in the BDC phase (BDC-7), on days 29 and 58 during HDBR (HDBR29, HDBR58, respectively), and 10 days post HDBR to assess recovery (R + 10). The measures reported in this study were acquired in tandem with those in our previous AGBRESA reports (34, 83) and overlap with our ongoing NASA supported flight and prior bed rest studies (26, 27, 36–40, 42–44, 79).

### 2.2 Dual-tasking

**Figure 2:**
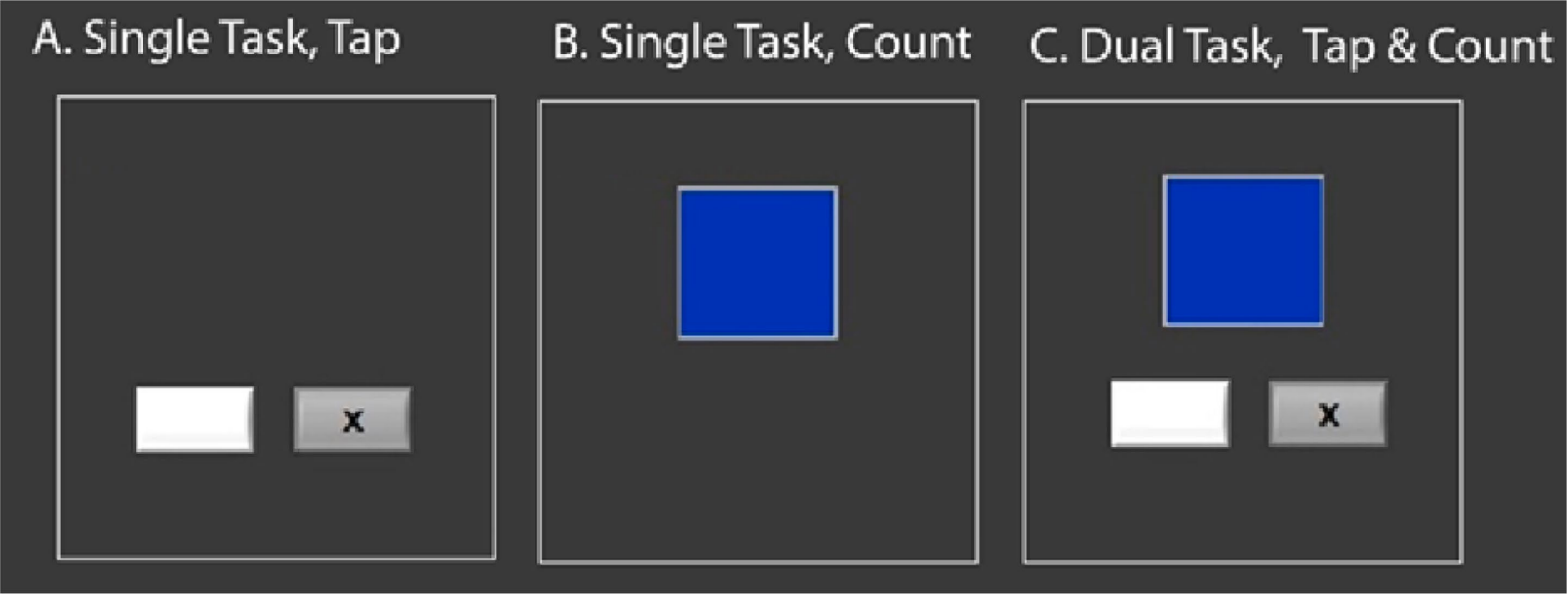
Cognitive-Motor dual-task. The gray boxes with a black X alternate rapidly, and the participant is required to hit a button on a left or right button box, corresponding with the side on which the x flashes. The blue box changes color rapidly; participants are instructed to count, and remember for the duration of the task, how many times the box changed to blue. They perform each as a single task, and also simultaneously under dual-task conditions.

During each of the data collections, participants performed a cognitive task, a motor task and both combined for dual tasking while in a Siemens 3T Magnetic Resonance Imaging (MRI) scanner. During the cognitive task participants were instructed to watch a small box that rapidly changed colors and to count the number of times that it turned blue. They were instructed to remember that count over the 4 trials (two as single task, two as dual task) and verbally report following all trials. The box changed colors at a rate of 3 Hz, with a low appearance rate of the blue color (1–3%), requiring sustained attention and oddball detection. During the motor task, participants were instructed to watch two small boxes that were side by side. When an “X” appeared in either box, they were to respond by pressing the corresponding button (i.e., left box, left button) on an MRI safe button box with either the left or the right index finger. The stimulus was presented randomly, with an inter-stimulus interval of 800 ms. Participants performed each of these tasks twice under single tasking and twice under dual tasking. We calculated participants’ reaction time, accuracy and dual-task cost for both measures on each task. The behavioral task results have been reported previously (34); we include the results here for brain-behavior correlation analyses (Figure 2).

### 2.3 Spatial working memory

In addition to the cognitive-motor dual task, participants performed a spatial working memory task that we have previously used in HDBR campaigns (77) and in spaceflight (6, 24). Participants performed the task in an MRI scanner, allowing us to capture functional MRI (fMRI). In this task, participants are shown three solid circles that appear for 500 ms before disappearing for 3,000 ms, leaving the screen blank. Participants are instructed to connect the dots into the shape of a triangle before the dots appear again in different positions. Participants must then identify if these dots are in the same triangular configuration but rotated, or if they form an entirely different triangle. Participants performed this task in two runs of 30 trials each. For the control phase of the task, participants must simply identify if a singular dot (presented for 500 ms) appearing on the probe screen is in the same position as one of the three dots previously presented, with a 200 ms delay. Participants performed one, 40-trial run of the control phase. The primary outcome metric of this task was accuracy (deciding whether or not the three dots were in the same configuration at the probe stage). We acquired fMRI during this task while participants were in the HDBR position inside of the MRI scanner (laying head down tilt on a foam wedge, with the head supine inside the coil).

### 2.4 MRI parameters

Data were acquired at the: envihab facility in Cologne, Germany with a 3-Tesla Siemens Biograph MRI Scanner. We used a gradient echo T2*-weighted echo-planar imaging sequence with the following parameters to acquire fMRI data: TR: 2500 ms, TE: 32 ms, flip angle: 90°, FOV: 192 × 192 mm, matrix: 64 × 64, slice thickness: 3.5 mm, voxel size: 3 × 3 × 3.5 mm, 37 slices. We also acquired a T1-weighted gradient-echo pulse sequence with the following parameters: TR: 1.9 s, TE: 2.4 ms, flip angle: 9°, FOV: 250 × 250 mm, matrix: 512 × 512, slice thickness: 1.0 mm, voxel size: 0.49 × 0.49 × 1.0 mm, 192 slices. During the fMRI collection, subjects were still in the HDBR position laying upon a foam wedge, however the head was flat within the MRI head coil.

### 2.5 MRI pre-processing

Pre-processing and statistical analysis of the fMRI data were conducted using Statistical Parametric Mapping 12 (SPM12; version 7,219), MATLAB R2019a, Advanced Normalization Tools [ANTs; (46)] and FMRIB Software Library [FSL; (47)]. First, fMRI field maps were created to map and remove B0 inhomogeneities utilizing the FSL topup tool (47). Next, we corrected the images for slice timing and then realigned and resliced to the mean slice to correct for volume-to-volume head motion in SPM12. We used the Artifact Detection Tool (ART^1^) to perform further quality checks. We covaried out volumes with a motion threshold greater than 2.0 mm, and a global brain signal Z threshold equal to or greater than 9. We then used ANTs (46) to normalize the MRI images to the Montreal Neurological Institute 152 (MNI152) standard template in multiple steps. First, participant-specific T1 templates were created across time points using the AntsMultivariateTemplateConstruction.sh function; then, we created participant-specific mean functional templates using the same ANTs function. We co-registered each participant-specific functional template to their respective T1 template to acquire transform parameters using the AntsRegistration.sh function. Further, we normalized each participant’s specific template to the MNI152 standard space using AntsRegistrations.sh function as well. We then applied the resulting warp parameters to the participants’ fMRI runs using ANTs’ ApplyTransforms.sh function. We then used SPM12 to spatially smooth with an 8 mm full-width half-maximum three-dimensional Guassian kernel. This pre-processing procedure is the same as we have used in our past HDBR neuroimaging work (24, 27, 39).

### 2.6 Cerebellar pre-processing

We utilized special pre-processing methods for the cerebellum using a combination of the CEREbellum Segmentation [CERES; (80)] pipeline and the Spatially Unbiased Infratentorial Template [SUIT; (48, 49)] pipeline. We have used this pipeline previously to more clearly identify changes in the cerebellum [cf. (24, 27, 39)]. First, we ran the participant-specific T1 templates in the CERES pipeline to segment the cerebellum from the rest of the brain in each T1-weighted image. Then, we created a binary mask from the CERES native space output using ImCalc in SPM12 and used this to mask out the brain from the participant-specific T1 template in FSL. Then we used ANTs AntsRegistration.sh to transform the T1 cerebellar template to SUIT space. We then transformed the slice time corrected, realigned and resliced (as described above) functional runs into the participants’ T1 template space using AntsTransform.sh and masked with the binary cerebellar mask in FSL. Finally, we normalized the masked, functional runs into SUIT space using AntsApplyTransforms.sh. We then applied a 2 mm full-width half-maximum three-dimensional smoothing Gaussian kernel to SUIT space cerebellar images in SPM12. We chose the 2 mm kernel based on the small lobule size in the cerebellum; this is similar to other studies that have focused on cerebellar analyses (48–52).

### 2.7 Behavioral statistical analysis

The full statistical analysis is covered in-depth separately from here (34). To summarize, we used the R 3.6.1 (81) package nlme (82) to fit linear mixed models with restricted maximum likelihood to test for changes over time as participants enter HDBR. In each model, subject was considered as a random intercept to control for each participant’s different starting point. Mean centered age and sex were included as covariates within the statistical models.

### 2.8 Subject-level fMRI statistics

Single subject level brain activation for both tasks was calculated separately for the cerebellum and the whole brain. For dual-tasking, we calculated four statistical maps for each participant, at each time point, on a voxel-by-voxel basis with the following contrasts: (1) single motor task > rest, (2) single cognitive task > rest, (3) cognitive-motor dual task > rest, (4) dual task > both single tasks. For our SWM task, we assessed on a voxel-by-voxel basis the SWM task compared to rest (SWM > rest). As with our previous work (24, 27, 39, 79, 85), we used a first level masking threshold of –infinity to mask out non-brain regions using SPM12’s intracranial volume mask. With this, we were able to include all voxels in our first-level general linear models, as opposed to the SPM12 default where we would be limited to voxels with a mean value of ≥80% of the global signal.

### 2.9 Group level statistical analyses

To assess brain activation changes throughout HDBR we tested multiple group-level statistical models, described in detail below. First, we modeled hypothesis driven longitudinal changes where we predicted the AG group would reduce neural activation during task performance following entrance to HDBR and the CTRL group to increase neural activation; we also tested for the reverse contrast. Second, we tested all individuals as one group, in the case that AG did not show any effects. In this model, we tested for an overall increase in neural activation after all participants had entered the HDBR environment. We built these models using the Sandwich Estimate Toolbox for SPM12 [SWE; (53)]. SWE utilizes a noniterative marginal model to prevent within subject convergence problems that are inherent in longitudinal studies. This provides a superior analysis of longitudinal MRI data that can handle small subject samples sizes and missing data. We maintained the SwE defaults except for testing with 999 permutations for the non-parametric wild bootstrapping (54). Additionally, threshold free cluster enhancement was used [TFCE; (84)], as this approach does not require us to pre-specify an arbitrary cluster size and is more sensitive (55).

#### 2.9.1 Effects of AG versus control

To test whether the AG group differed from CTRL, we tested an *a priori* hypothesized weighted longitudinal model (Figure 1). We created longitudinal contrasts that included all 4 time points with each weighted to test for our hypothesized changes. We have used the same approach and weights in our previous AGBRESA investigations (83, 85) and similar approaches in other HDBR analog and spaceflight experiments (24, 27, 37–39, 43, 45, 79). The models reflect hypothesized linear changes, whether that be increases or decreases. Mean centered age and sex were included in the model as covariates. Significance was analyzed at a *p* < 0.05, family-wise error (FWE) corrected for multiple comparisons. For whole brain analysis, an explicit mask was used to run analysis only on gray matter. This mask was created through binarizing the Computational Anatomy Toolbox 12 [CAT12; (56, 57)] MNI-space gray matter template at a threshold of 0.1. The cerebellum was masked out with the SUIT.nii cerebellar template. Cerebellar analyses was conducted only on the cerebellum as discussed above in “Cerebellar Pre-processing” with no mask utilized; all other specifications were the same.

#### 2.9.2 Effects of HDBR

To assess the effects of HDBR on cognitive task function we utilized a similar approach as discussed above. However, here we pooled all subjects (AG and CTRL) into one large group. This allows us to search for brain regions that show a similar pattern of change for all participants, regardless of AG dosage.

#### 2.9.3 Brain and behavior change – change correlations

To assess the effects of AG on HDBR, we used a similar approach in brain-behavioral correlations as discussed above. However, here we assessed the change in cognitive behavioral performance between BDC-7 and HDBR58 and the changes in brain activation at the same time points to assess for overall changes that may occur due to 60 days of HDBR. We conducted this analysis with all participants pooled into one group.

## 3 Results

To assess significance, we used a family wise error (FWE) corrected *p* value of FWE < 0.05 and voxel extent of k˃10. Our initial analyses identified no significant or trending differences in brain activation or behavior between the two AG groups (intermittent versus continuous). We therefore pooled them together to increase statistical power.

### 3.1 Behavior

As mentioned previously, full details of the behavioral performance are provided in a previous publication (34). We identified no significant group by time interactions in the motor single task (tapping), the cognitive single task (counting), dual tasking, dual task cost nor SWM performance. We include the data here to examine relationships between individual differences in brain and behavior changes.

### 3.2 HDBR + AG

To assess the counter-active effects of AG on HDBR, we tested our hypothesis that those that did not receive AG daily would have greater changes in brain activity with HDBR, whereas the AG group would either maintain or decrease their activation. We identified no brain regions that followed this hypothesized trend in either dual-task or SWM brain activity that met our pre-determined alpha level of FWE < 0.05. Further, there were no trending effects approaching significance.

### 3.3 HDBR

**Figure 3:**
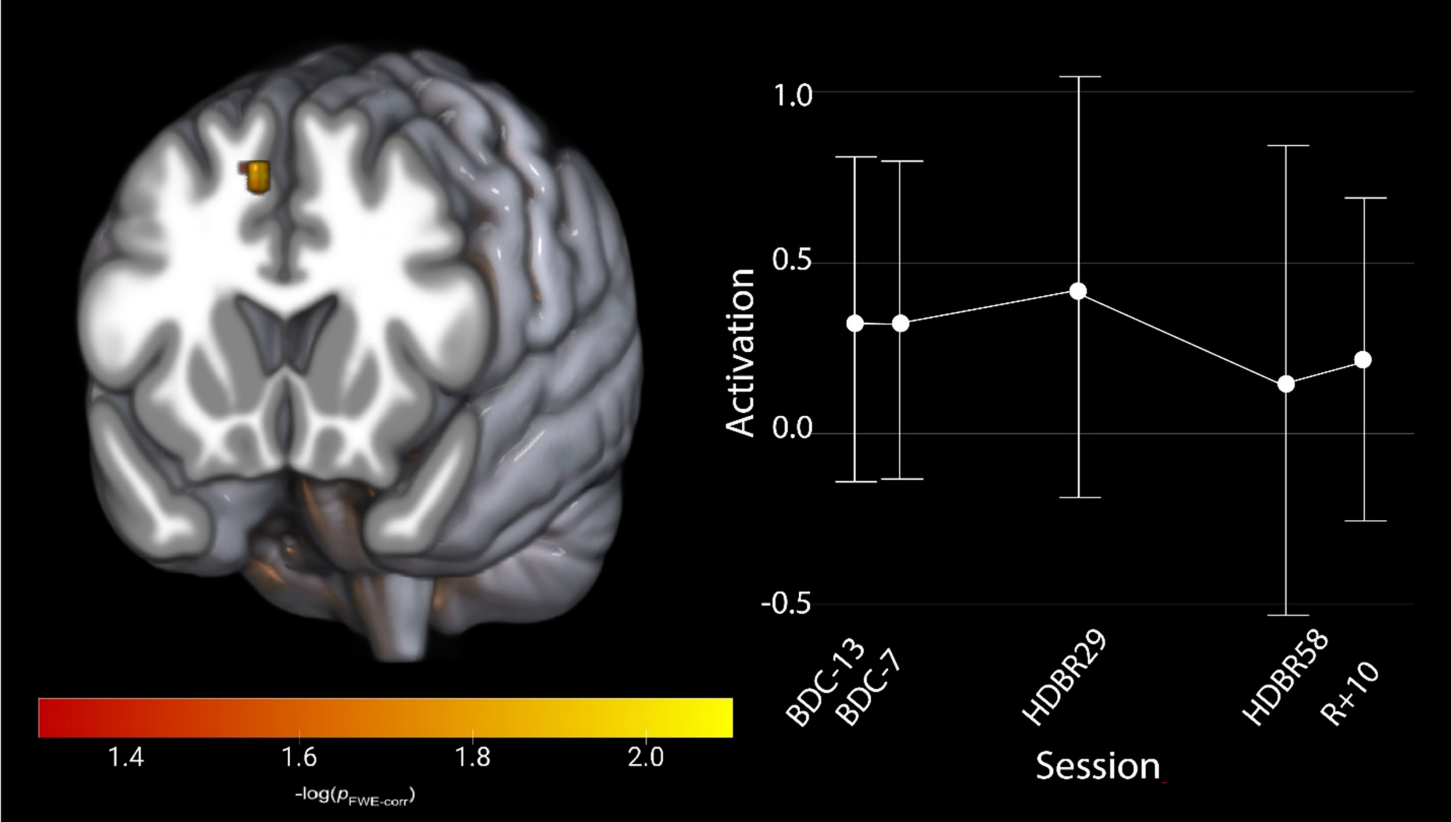
Activation changes across HDBR in all participants during cognitive-motor dual-tasking, where all participants show an increased activation shortly upon entering HDBR, that decreases by HDBR58. The activation profile for the right pre-supplementary motor region is shown for illustration. Results are overlaid onto the MNI standard template. pFWE-corr, yellower colors represent smaller *p*-values.

To assess the effects of HDBR on cognitive-motor dual-tasking, regardless of the effects of AG, we evaluated our hypothesis that activation would increase in task relevant brain regions after entering the HDBR environment, potentially reflecting decreased neural efficiency. We identified one cluster (FWE < 0.05, Table 1) that displayed a significant effect of HDBR during dual task performance. All participants increased brain activation within the right pre-supplementary motor area (58) following their entrance into HDBR; this change recovered over time across the 60 days spent in the HDBR environment (Figure 3). There were no other identified brain changes in either single task or dual task cost.

**Table 1:** Brain changes that display longitudinal changes due to HDBR during dual-tasking.

Brain region	FWE level		MNI coordinates (mm)		
	*p*_FWE-corr_	Extent (k_E_)	*X*	*Y*	*Z*
R pre-supplementary motor	0.002	10	14	4	50

Brain regions that showed longitudinal changes due to HDBR in dual-tasking. Significance set at *p*_FWE-corr_<0.05, FWE corrected. Clusters were labelled based on the AAL3 atlas. R = Right.

**Figure 4:**
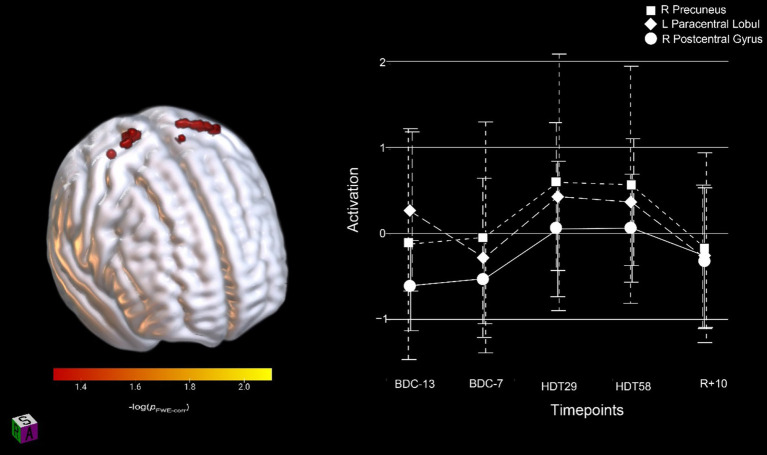
Activation changes across HDBR in all participants during SWM; all participants showed increased activation shortly upon entering HDBR that was maintained throughout HDBR. The activation profile for the right precuneus, left paracentral lobule and right postcentral gyrus regions are shown. Results are overlaid onto the MNI standard template. pFWE-corr, yellower colors represent smaller *p*-values.

To assess the effects of HDBR on SWM, in the absence of counter-active AG effects, we assessed all participants’ neural activation in a single group. We identified three significant clusters (FWE < 0.05, Table 2) that showed an increase in activation following entrance to the HDBR environment that continued to increase throughout the HDBR period before returning to baseline levels in the recovery phase (Figure 4).

**Table 2:** Brain changes that display longitudinal changes due to HDBR during spatial working memory.

Brain region	FWE level		MNI coordinates (mm)		
	*p*_FWE-corr_	Extent (k_E_)	*X*	*Y*	*Z*
R precuneus	0.043	97	12	-44	68
R postcentral gyrus	0.048	10	26	−42	60
L paracentral lobule	0.044	80	−16	−30	76

Brain regions that showed longitudinal changes due to HDBR during spatial working memory. Significance set at *p*_FWE-corr_<0.05. Clusters were labelled based on the AAL3 atlas. L = Left; R = Right.

### 3.4 Brain and behavior correlations

**Figure 5:**
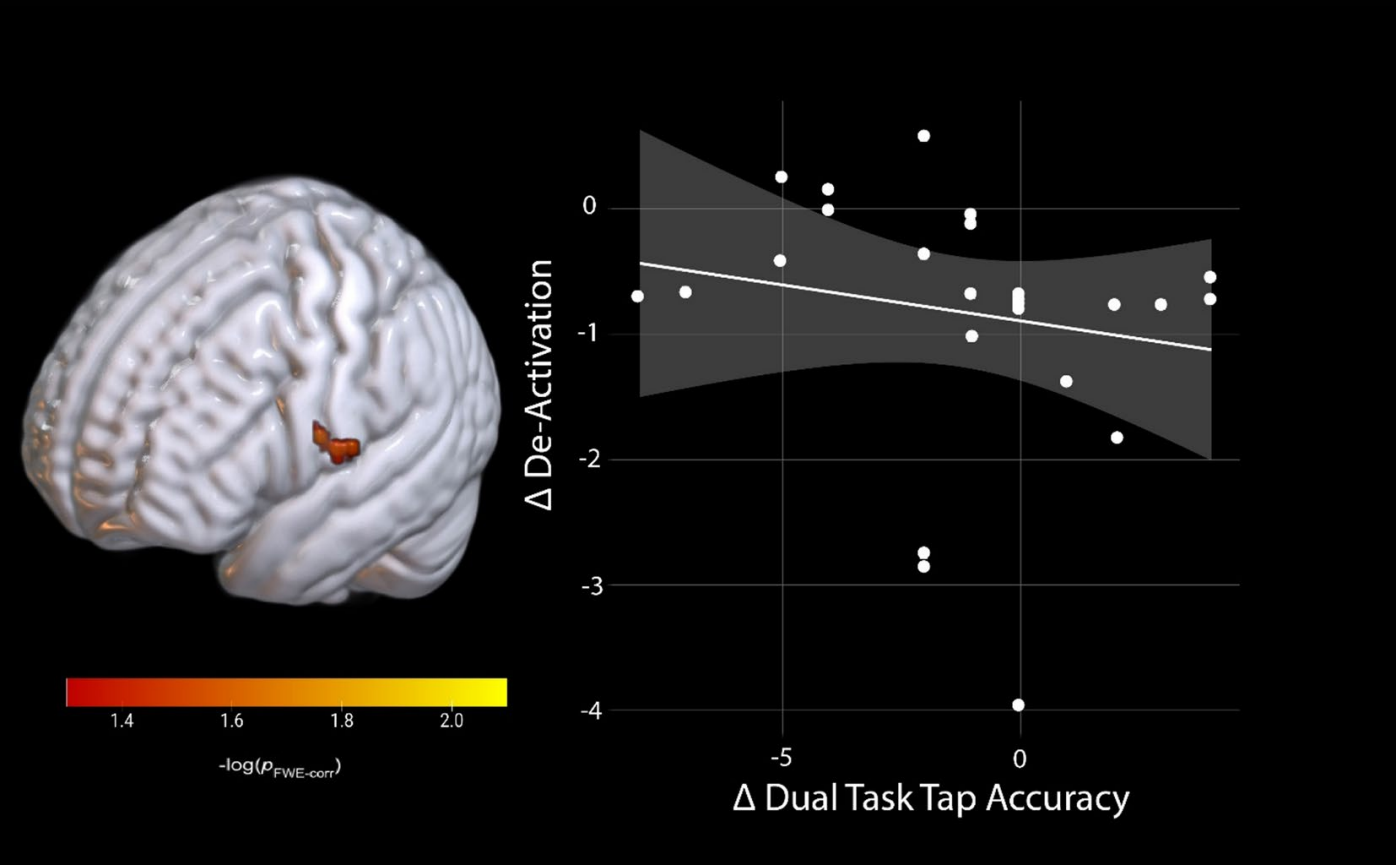
Brain and behavior change-change correlation of dual-task tapping. Change on the De-activation scale indicates a lower activation during late-HDBR compared to pre-HDBR, and change on dual-task tap accuracy scale indicates lower accuracy during late-HDBR compared to pre-HDBR. pFWE-corr, brighter colors represent smaller *p*-values.

We conducted brain-behavior change-change correlations with all participants in one group (AG and Control). In the SWM task, we did not identify any significant brain-behavior correlations from BDC-7 to HDBR58. For dual-tasking, we also identified no significant relationships between brain activity changes and changes in the cognitive single or cognitive dual task accuracy. However, for the dual task tapping accuracy, we identified a significant relationship between activation changes in the left postcentral gyrus (*p*_FWE_ < 0.05; Table 3) and dual task tap accuracy. Individuals who increased activation (reduced their de-activation) the most had the smallest decrease in motor tapping accuracy from pre-HDBR to late-HDBR (HDBR day 58, Figure 5).

**Table 3:** Brain and behavior change-change correlation during dual-tasking.

Brain region	FWE level		MNI coordinates (mm)		
	*p*_FWE-corr_	Extent (k_E_)	*X*	*Y*	*Z*
L postcentral gyrus	0.025	48	−59	0	20

Brain regions that showed change-change correlations with behavior. Significance set at *p*_FWE-corr_<0.05. Clusters were labelled based on the AAL3 atlas. L = Left.

## 4 Discussion

We investigated whether AG would mitigate HDBR effects on brain changes that occur during cognitive-motor dual-tasking or during SWM task performance. We observed no effect of AG on brain changes in single tasking, nor dual-task cost. We identified an initial increase followed by decreased activation within the right pre-supplementary motor cortex that was specific to the HDBR phase during dual-tasking (Figure 3). We also identified that during the SWM task, the right precuneus, the right postcentral gyrus and the left paracentral lobule increased activation once participants had entered the HDBR environment; this effect remained constant throughout HDBR and was resolved by 10 days post-HDBR. Finally, we identified a dual-tasking brain-behavior change-change correlation in the left postcentral gyrus, in which individuals that increased their brain activation the most maintained their dual-task tapping performance relative to baseline. Taken together, we interpret these findings to reflect that 30 min of AG daily has minimal effects on brain changes underlying cognitive function during HDBR, at least for the duration and dosage used in the AGBRESA campaign.

While we did not identify any counter-active effects of AG on dual-tasking brain activation, we did see increased activation in the right pre-supplementary motor area (preSMA) for the combined groups during dual tasking performance that was specific to the HDBR period (Figure 3). This increase in activation subsided by the end of HDBR, suggesting adaptation occurred over time in HDBR. We observed similar effects in a different HDBR study (45), suggesting that this region may be particularly affected by HDBR. The preSMA has been argued to be important for movement control, particularly for complex actions and cognitive control of movement (59). This region also plays a critical role in stopping action (60–62), suggesting that HDBR may affect cognitive-motor interactions. We further identified a brain-behavior change-change correlation during dual-tasking where those that decreased their activation within the left postcentral gyrus the most maintained their tapping performance relative to baseline at the end of HDBR (Figure 5). The postcentral gyrus comprises of the somatosensory cortex, the primary region associated with sensory perception from the body (63). Interestingly, this correlation would suggest that those that were the most able to decrease activation in a region heavily involved in sensory perception region maintained their pre-HDBR motor performance. This may reflect sensory reweighting. It is not directly clear how this result relates to performance, however, since the cluster is in a region that is substantially inferior to the hand knob location.

Similar to cognitive-motor dual-tasking, AG had no effects on SWM performance and functional brain activation. All participants (AG and CTRL) increased activation in various brain regions during spatial working memory task performance after they entered the HDBR environment. Specifically, we identified increased activation within the right precuneus, the right postcentral gyrus and the left paracentral lobule (Figure 4) that persisted throughout HDBR and resolved by 10 days after participants exited the HDBR environment. The precuneus has been shown to play a role in visuo-spatial and higher cognitive function (64). The precuneus has also been argued to serve as a major node in various functional brain networks, including two subnetworks of the default mode network (65). The default mode network is heavily involved in introspection and rumination, and is considered an anti-task network, exhibiting decreased activity when participants perform a task relative to rest (66). This increased activation of the precuneus could indicate difficulty with network switching between task and default mode networks; similar effects have been seen in older adults (67). The postcentral gyrus receives somatosensory projections via the thalamus (63), as discussed above. The somatosensory cortex is primarily lateralized, with the left hemisphere receiving inputs from the right side of the body (68, 69). Here we saw increased activation in the right hemisphere, while participants were using their right hand. This implies a compensatory response, as compensation typically involves recruitment of bilateral brain regions (70). Due to the reduced somatosensory inputs that occur in HDBR (with the body largely unloaded), bilateral recruitment of the postcentral gyrus may increase the gain of somatosensory signaling. We also observed increased activation in the left paracentral lobule, another region which has been shown to process somatosensory inputs (71). This would also align with previous findings showing that HDBR results in increased functional connectivity between the posterior parietal cortex and somatosensory cortices, and AG was associated with decreased connectivity between these regions, suggesting compensatory responses in somatosensory processing due to HDBR (86). Further, those in the AG group that decreased their connectivity the most had minimal mobility declines from pre-to post-HDBR (86). Overall, these changes in brain activation patterns suggest that the HDBR environment is making this SWM task more difficult to perform, resulting in recruitment of additional brain regions. It is interesting that these are largely in somatosensory cortical regions rather than in frontoparietal areas, typically engaged for SWM tasks (72).

These cognitive assessments were included as part of a large HDBR campaign with funding from American, German and European space associations (NASA, DLR & ESA; AGBRESA). The overall aim was to determine whether AG would serve as an effective integrated countermeasure for mitigating many HDBR changes that occur. In this campaign, another group reported that HDBR resulted in cognitive performance declines, with no benefit of AG (28). We found largely similar results, with the exception of cognitive task performance during AG centrifugation. Those that received AG had better cognitive performance on the Paced Auditory Serial Addition Task (PASAT) (34) than those that performed it during HDBR alone. This, taken in conjunction with the present findings, suggests that AG does not have a strong impact on cognition, but it could be useful for cognitive tasks performed during centrifugation.

The literature suggests that the primary advantages of AG are benefits to sensory and motor behavior. We have shown that AG can increase neural efficiency in visuomotor rotation tasks (83); further, we observed that those who decreased brain activity the most during task performance showed the least HDBR associated performance declines. We also found that the AG group showed greater decreases in resting state functional connectivity with the posterior parietal cortex, a major hub of sensory integration, than controls (86). Further, those in the AG group that showed the largest decreases in connectivity had minimal mobility declines from before to after HDBR (86). We have also shown that HDBR results in balance declines; however, those in the AG group had less declines, as evident by the large effect size even though not statistically significant (34). Thus, while AG ameliorates some of the sensorimotor effects of HDBR, the dosage of 30 min daily may be insufficient to see counter-active effects in all aspects of cognition. Moreover, the centrifugation velocity resulted in 1 Gz at the heart, but only 0.3 Gz at the level of the head. Increasing the dosage to reach levels of 1Gz at the head may result in more pronounced effects of AG on cognition and the brain. However, this would also increase Gz at the heart and the feet and may not be optimal for function in those areas. Other doses/durations of AG may have greater effects on neurocognitive function. It should be kept in mind, however, that findings in HDBR do not completely map onto those observed in the microgravity environment [for review see: Barkaszi et al. (73)].

The sample size of this study is limited, particularly if the AG groups are kept separate. This makes it difficult to identify more nuanced group differences, particularly when investigating cognitive changes with HDBR as effect sizes are generally not large. Moreover, the time spent in HDBR for this campaign was 60 days, whereas a typical mission to the ISS lasts approximately 6 months. We do not currently understand whether or how the duration of HDBR scales to the duration of microgravity, particularly in terms of neurocognitive changes. Sixty days of HDBR does result in many similar effects to microgravity on the musculoskeletal and cardiovascular systems, though. Moreover, the centrifugation velocity in this study resulted in 1 Gz at the CoM, approximately 2 Gz at the foot, and approximately one third Gz at the head. It is possible that the lack of effects here are due to the level of AG at the head not being sufficient to mitigate structural and functional brain changes from HDBR. Future studies should investigate greater levels of Gz at the head to potentially increase neurological benefits. It is also important to note, that the HDBR environment may increase levels of physiological stress, such as with sleep disturbances that could have impacts brain connectivity and cognitive task performance (74, 75). Ultimately, while AG does not appear to have a clear effect on cognitive function in HDBR, further examination is warranted with a greater emphasis on larger sample sizes and varying AG doses.

Here, we investigated whether AG is an effective countermeasure for HDBR induced performance changes in cognitive-motor dual tasking and spatial working memory. We identified no effects of AG on either task, instead finding increased activation in somatosensory brain regions for all participants after they entered the HDBR environment. Further, we identified a brain-behavior correlation in dual-tasking where those that decreased their activation the most in the left postcentral gyrus had the highest level of performance. Overall, these findings would suggest that AG, as applied in this study, is not sufficient to counteract functional brain changes that occur in HDBR, and possibly spaceflight.

## Data Availability

The raw data supporting the conclusions of this article will be made available by the authors, without undue reservation.

## Footnotes

^1^ https://www.nitrc.org/projects/artifact_detect/
